# Graphical representation of quality indicators based on medical service ontology

**DOI:** 10.1186/2193-1801-2-274

**Published:** 2013-06-23

**Authors:** Osamu Takaki, Izumi Takeuti, Koichi Takahashi, Noriaki Izumi, Koichiro Murata, Mitsuru Ikeda, Koiti Hasida

**Affiliations:** School of Knowledge Science, Japan Advanced Institute of Science and Technology, 1-1 Asahidai, Nomi, 923-1292 Japan; Social Intelligence Technology Research Laboratory, National Institute of Advanced Industrial Science and Technology, Tsukuba, 305-8568 Japan; School of Medicine, Kitasato University, Sagamihara, 252-0373 Japan

**Keywords:** Quality indicator, Medical service, Quality assessment, Ontology, Graph representation

## Abstract

For recent years, it has grown importance to evaluate medical service qualities of medical staffs and/or hospitals by using quality indicators. This paper introduces a representation system QI-RS of quality indicators. By using QI-RS, one can define quality indicators that satisfy understandability and formality, where “understandability” means that one can understand the calculation formula of a quality indicator easily and correctly, while “formality” means that the formula can be calculated to obtain the values of the indicator based on databases in a coherent manner.

## Introduction

Quality indicators are measures of medical service quality that are represented by numerical values. A quality indicator consists of a *name* (or a *label*) and a *calculating formula*. For example, “fracture rate among inpatients aged 75 or older” is the name of a quality indicator, and its calculating formula is given as follows.

**Calculating Formula (CF1)** (Nihon Hospital Organization 2009). The data of the quality indicator above is obtained by calculating the proportion of the following values.

-  
*Numerator.* the number of inpatients that satisfy the condition defined in the denominator and that broke their bones in a hospital and received treatment for the fractures.
-  
*Denominator.* the number of patients that were hospitalized for three days or more, and aged 75 or older when they were admitted into the hospital.

The value that is obtained from a quality indicator by using the calculating formula and data in a hospital (or hospitals) is called “the value of a quality indicator (in a hospital (or hospitals))” or “the data of a quality indicator (in a hospital (or hospitals))”.

It is important to evaluate medical service qualities of medical staffs and/or hospitals by using quality indicators (Donabedian 1980; Ito 2003; Mainz 2003). Moreover, there are some groups of hospitals that compare their service quality by using their quality indicators (Hata et al. 2009; Mainz et al. 2004; OECD 2006).

However, to compare medical service quality by using quality indicators, one has to define quality indicators that satisfy that others can understand the contents of the indicators, where “to define a quality indicator” means “to explicitly describe a calculating formula of a quality indicator”. For example, to understand the calculating formula CF1 correctly, one has to make clear at least the following two points:

-  What data should be a fact that an inpatient broke his/her bone checked by?
-  What is the definition of “treatment of bone fracture”?

To this end, it is significant to establish a way to unify a vocabulary of quality indicators and their interpretation including their whole structures. In such a case, natural language is not suitable for defining quality indicators with ensuring unambiguity and reasonability of their definitions. Moreover, to calculate the results of the quality indicators from databases in hospitals, one has to calculate the results in a coherent manner. Thus, quality indicators should be defined to be machine readable.

This paper introduces a representation system QI-RS of quality indicators. By using QI-RS, one can define quality indicators that satisfy understandability and formality, where “understandability” means that one can understand the calculation formula of a quality indicator easily and correctly, while “formality” means that the formula can be calculated to obtain the result of the indicator from databases in a coherent manner. QI-RS consists of the three main components: (i) Medical Service Ontology (MSO), (ii) object graphs, and (iii) quantifying concepts. MSO is defined as a vocabulary of quality indicators. An object graph is a graph whose nodes and edges are labeled by concepts or values and properties in MSO. While an object graph denotes a target of quantification, a quantifying concept denotes a way to quantify a concept that is denoted by an object graph.

In the previous papers (Takaki et al. 2012a; Takaki et al. 2012b), the authors introduced MSO, object graphs and quantifying concepts. However, they have not yet made clear how to construct them to define quality indicators and basic theory by which MSO is defined definitely. Moreover, since object graphs were defned by inductive definition on the structures of graphs in (Takaki et al. 2012a; Takaki et al. 2012b), the definition of object graphs was complicated and it often prevented one from developing quality indicators in a simple manner. Thus, this paper introduces basic theory of objet graphs, and definition of quality indicator graphs consisting of object graphs and quantifying concepts. By virtue of the basic theory of object graphs, it is simpler to develop quality indicators by using small subgraph (the method is called “incremental construction of quality indicators“).

In the last half of this paper, to evaluate expressiveness of QI-RS, the authors re-define existing quality indicators in (Fukui 2009; Nihon Hospital Organization 2009; OECD 2006) by using QI-RS. This paper also explains how to define a complex quality indicator with QI-RS, by using an existing quality indicator in (Nihon Hospital Organization 2009).

The remainder of this paper is organized as follows. Section 2 briefly explains an overview of a framework to develop quality indicators and to calculate their values based on medical databases. Section 3 explains the outline of QI-RS. Section 4 explains graph-based syntax of quality indicators in QI-RS. Section 5 explains MSO. Section 6 explains quantifying concepts that are used in quality indicators in QI-RS. Section 7 explains graph-based representation of quality indicators. Section 8 shows an evaluation of expressiveness of QI-RS via re-defining of existing quality indicators in QI-RS. The last two sections are related works and conclusions, respectively.

## QI-framework

**Figure 1:**
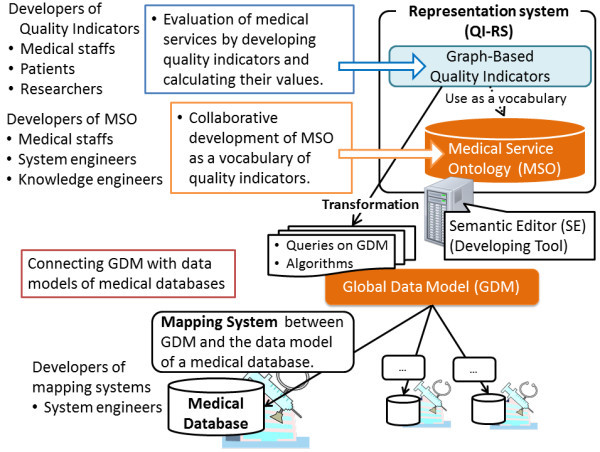
**Overview of QI-framework.**

Though the purpose of this paper is to introduce a representation system QI-RS of quality indicators, this section briefly explains a framework to develop quality indicators and to calculate their values based on medical databases before entering upon a discussion of QI-RS. The framework is called as QI-FW. QI-FW consists of (1) QI-RS, (2) Medical Databases in hospitals, and (3) Mapping Systems (Figure 1).

Developers of quality indicators, such as medical staffs, patients and researchers, use QI-RS to develop quality indicators. QI-RS is a graph-based representation system which includes MSO as its vocabulary. MSO are developed and improved in collaborative works that are achieved by medical staffs, system engineers and knowledge engineers. Here, system engineers are staffs in hospitals that employ QI-FW who well know data models on medical databases. Compositions of MSO will be discussed in Section 5.

As a tool that technically assists developing and improving MSO, *Semantic Editor* (SE) (Hasida 2012) is employed. SE is an environment for developments of RDFS-based ontologies. When one defines a concept or a property in an ontology by using SE, it is defined based on a basic class of RDFS (Resource Description Framework Schema) (W3C 2004). Several main properties in SE such as subclass property will be briefly explained in Section 4.2.

Concepts and properties in MSO are automatically translated to entities or terms in entities in Global Data Model (GDM), that is a virtual data model, under certain rules. According to the translation, a quality indicator *Q* in QI-RS is translated to a data (*Q*_*1*_,…, *Q*_*n*_, *A*) consisting of queries *Q*_*1*_, …, *Q*_*n*_ on GDM and an algorithm *A* on the data obtained from *Q*_*1*_, …, *Q*_*n*_. On the other hand, system engineers who well know a medical database develop and improve a mapping system between GDM and the data model of the database. Through the mapping system, the data (*Q*_*1*_,…, *Q*_*n*_, *A*) above is translated to data consisting (*Q*_1_^*^,…, *Q*_n_^*^, A^*^) of queries on the data model and algorithms on the data obtained from the queries. By the data (*Q*_1_^*^,…, *Q*_n_^*^, A^*^), a user can automatically obtain the values of *Q* in the medical databases.

When a user of QI-FW develops a quality indicator by using QI-RS, he/she employs MSO as a vocabulary, where the vocabulary includes not only words but also relations of words. On the other hand, system engineers determine how to translate concepts and properties of MSO by developing a mapping system between GDM and the data model of their database. Thus, the interpretation of the vocabulary to develop quality indicators is made clear, and the interpretation can be easily identified by users of QI-FW.

The authors interviewed system engineers in a university hospital who managed medical databases, developed quality indicators and calculate their values based on the medical databases about how to develop quality indicators and how to calculate their values. Then, the authors found that there was a single word that had multiple meanings while there are multiple words that shared a single meaning among different hospitals or difference medical databases. Uses of QI-WF can share quality indicators by using mapping systems that make each word have a single meaning for the users even if the uses use difference medical databases in different hospitals. Moreover, a user of QI-FW can uniquely define a special word such as “hospital stays of aged patients” and register the special word in QI-RS. Then, other uses can reuse the special word while checking the definition of the word easily (see also Section 8.1).

## Outline of QI-RS

The representation system QI-RS is developed based on an idea to regard a quality indicator as a combination of a target of quantification and a way to quantify the target and to develop the target and the way independently. For example, the calculating formula CF1 in Section 1 is regarded as a combination of the following components.

1. The set of hospital stays of patients aged 75 or over in which they broke their bones and received treatments of bone fractures.
2. The rate of the number of the hospital stays above over that of hospital stays of patients aged 75 or over.

The components above indicate that CF1 is determined by defining what the rate is or how the rate should be calculated as well as what the target of calculation is. Thus, the target of the calculation or the quantification is represented as a graph called an *object graph*, while the way to calculate or quantify object graphs is represented by a concept called a *quantifying concept*. Moreover, object graphs are constructed based on *Medical Service Ontology* (MSO).

In the following sections, a fundamental theory of QI-RS is introduced, where ontologies, ontology-labeled graphs and object graphs, which are special ontology-labeled graphs, are defined. Then, MSO (Section 5), quantifying concepts (Section 6) and finally graph-based representations of quality indicators called *quality indicator graphs* (Section 7) are defined. Quality indicator graphs are combinations of object graphs and quantifying concepts.

## Fundamental theory of graphical representation for quality indicators

### Graph

**Definition (Graph)** A quadruple (*N*, *E*, *src*, *tagt*) is a *graph* if the followings hold.

1. *N* and *E* are finite sets.
2. *src* and *tagt* are functions of *E* into *N*.

Definition (Terminologies on Graphs)

1. An element of *N* is called a *node*.
2. An element of *E* is called an *edge*.
3. If *src* (*e*) = *n* and *tagt*(*e*) = *n’*, then it is said that *e* is an *edge from n to n*’ and described by *e*: *n* → *n*’.

**Definition (Path)** A graph *G* = (*N*, *E*, *src*, *tagt*) is given. A sequent of edges (*e*1, *e*2, …, *em*) is a *path* from *n* ∈ *N* to *n*’ ∈ *N* in *G* if there are nodes *n* = *n*_0_, *n*_1_, …, *n*_*m*_ = *n*’ which satisfies the following property:

- For each *e*_*i*_ ∈ {*e*_1_, *e*_2_, …, *e*_*m*_}, either *e*_*i*_: *n*_*i*-1_ → *n*_*i*_ or *e*_*i*_: *n*_*i*_ → *n*_*i*-1_.

The empty sequence is also a path. Thus, for each *n* ∈ *N*, the empty sequence is a path from *n* to *n*.

**Definition (Terminology on Paths)** A path *p* = (*e*1, *e*2, …, *em*) *passes through* a node *n* ∈ *N* if either *src*(*ei*) = *n* or *tagt*(*ei*) = *n* for some *ei* ∈ {*e*1, *e*2, …, *em*}.

**Definition (Connectivity)** For a graph *G* = (*N*, *E*, *src*, *tagt*), two nodes *n*, *n*’ ∈ *N* are *connected* in *G* if there exists a path from *n* to *n*’. A graph *G* is connected if any two nodes are connected in *G*.

**Definition (Subgraph)** A pair (*N*, *E*) is a *subgraph* of a graph (*N*0, *E*0, *src*, *tagt*) if the following properties hold:

1. *N* ⊂ *N*_0_, *E* ⊂ *E*0.
2. For *e* ∈ *E*, both *src*(*e*) ∈ *N* and *tagt*(*e*) ∈ *N* hold.

**Remarks on Subgraphs** A graph *G* = (*N*_0_, *E*_0_, *src*, *tagt*) is given. If (*N*, *E*) is a subgraph of *G*, then (*N*, *E*, *src*|*E*, *tagt*|*E*) is a graph, where *src*|*E* and *tagt*|*E* are restrictions of *src* and *tagt* over *E*, respectively. A graph (*N*, *E*, *src*|*E*, *tagt*|*E*) is often abbreviated to (*N*, *E*), and *src*|*E* and *tagt*|*E* to *src* and *tagt*, respectively.

### Ontology and ontology-labeled graph

**Definition (Ontology)** A septuplet (*Conc*, *Val*, *Prop*, *Attr*, *src*, *tagt*, ≦) is an *ontology* if the following properties hold:

1. *Conc*, *Prop* and *Attr* are finite sets such that *Attr* ⊂ *Prop*.
2. *Val* is a set.
3. ≦ is a partial order over *Conc*∪*Val* such that each element in *Val* is maximal w.r.t. ≦.
4. *src* and *tagt* are functions of *Prop* into the power set of *Conc*∪*Val*.
5. For an element *P* of *Prop*, the return values *src*(*P*) and *tagt*(*P*) are upward closed w.r.t. ≦, that is, the following properties hold:
  For each *N, N’* ∈ *Conc*∪*Val* and each *P* ∈ *Prop*, if *N* ∈ *src*(*P*) and *N*≦*N’*, then *N’* ∈ *src*(*P*).
  For each *N, N’* ∈ *Conc*∪*Val* and each *P* ∈ *Prop*, if *N* ∈ *tagt*(*P*) and *N*≦*N’*, then *N’* ∈ *tagt*(*P*).

**Definition (Terminologies on Ontologies)** An ontology *O* = (*Conc, Val, Prop, Attr, src, tagt,* ≦) is fixed.

1. An element of *Conc* is called a *concept name* or simply a *concept*.
2. The notation *NL*_*O*_ denotes the set *Conc*∪*Val*. *NL*_*O*_ is sometimes abbreviated to *NL*.
3. An element of *NL* is called a *node label*.
4. An element of *Val* is called a *value*.
5. An element of *Prop* is called a *property name* or simply a *property*.
6. An element of *Attr* is called an *attribute name* or simply an *attribute*.
7. For *C* ∈ *Conc* and *A* ∈ *Attr*, if *C* ∈ *src*(*A*), then it is said that *C has an attribute A* and that *A* is an *attribute of C*.
8. The notation *attr*_*O*_ (*C*) denotes the set {*A* ∈ *Attr| C* ∈ *src*(*A*)}. *attr*_*O*_(*C*) is sometimes abbreviated to *attr*(*C*).

**Example of an Ontology (Simple Medical Service Ontology).** As a small example of an ontology, *Simple Medical Service Ontology (SMSO)* is defined, as follows.

SMSO consists of the following components:

1. *Conc*:={[human], [patient], [medical service], [diagnosis], [operation], [abdominal operation], [disease], [date]}.
2. *Val:*={[stomach cancer], [bowel cancer], [lung cancer], [1950/11/10], [1975/5/8], [2012/10/25], [2012/10/30], [2012/12/3]}.
3. *Prop*:={[to who], [after], [before], [birth date], [when], [result]}.
4. *Attr*:={[birth date], [when], [result]}.
5. SMSO has a special partial function *typ* of *Conc* into the power set of *Val*, which is defined as follows:
  *typ*([disease]):={[stomach cancer], [bowel cancer], [lung cancer]}.
  *typ*([date]):={[1950/11/10], [1975/5/8], [2012/10/25], [2012/10/30], [2012/12/3]}.
  For any other concept name, the value of *typ* is not defined.
6. An partial order ≤ is defined, as follows:
  *typ*([disease]):={[stomach cancer], [bowel cancer], [lung cancer]}.
  For each *C*, *C’*∈*Conc*, *C* ≤ *C’* iff there exists an edge from *C’* to *C* that is labeled by “subClassOf” in Figure 2.

**Figure 2:**
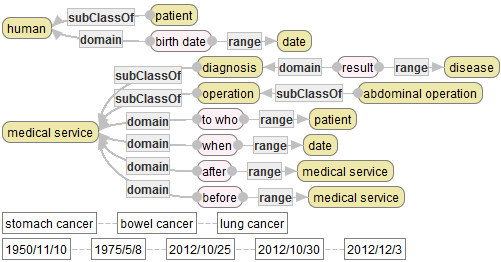
**Simple medical service ontology.**
  For each *C* ∈ *Conc* and *v* ∈*Val*, *C* ≤ *v* iff there exists C’ such that *v* ∈ *typ*(*C’*) and *C* ≤ *C’*.
  For each *v, v’* ∈*Val*, *v* ≤ *v’* iff *v* = *v’*.
7. For each property *R* in SMSO, *src*(*R*) and *tagt*(*R*) are indicated by the labeled edges “domain” and “range” in Figure 2, respectively. For example, the values of *src* and *tagt* at the property name [to who] are defined respectively, as follows:

To enable developing SMSO by using Semantic Editor (SE), the following supplemental definitions are introduced. SE has RDFS as basic language of ontologies. Let consider an ontology *O* = (*Conc, Val, Prop, Attr, src, tagt,* ≦). Then, concepts and properties of *O* can be defined as elements of the class “Class” and those of another class “Property” of RDFS, respectively. Moreover, the partial order ≦ on concepts can be regarded as a basic property in RDFS that is called “subclass-of” property, as follows: for concepts *C* and *D*, *C*≦*D* if there exists a subclass-of property from *D* to *C*, which is often said that “*D* is a subclass of *C*”. Furthermore, *src* and *tagt* can be defined by basic properties that are called “domain” and “range” properties in RDFS, as follows: for a concept *C* and a property *P*, *C* ∈ *src*(*P*) (or *C* ∈ *tagt*(*P*)) if there exists a domain property (a range property, respectively) from *P* to *C*, which is often said that “*P* has *C* as its domain (range, respectively)”. To summarize the points above, one obtains the following table (Table 1).

**Table 1:** **Predicates in the definition of ontologies and representations of predicates in SE**

	Predicates in the definition of ontologies in this paper	Representations of predicates in SE
*“D* is a subclass of *C*”	C≦D	*C* ← subclass-of-*D*
“A property *P* has a concept *C* as its domain.”	C ∈ src(P)	*C* ← domain-*P*
“A property *P* has a concept *C* as its range”	C ∈ tagt(P)	*C* ← range-*P*

The following figure depicts SMSO that is developed in SE.

In Figure 2, yellow (highly-colored) rotundate rectangles describe concepts in SMSO, while pink (softly-colored) rotundate rectangles describe properties in SMSO. Moreover, arrows with label “subClassOf” describe the partial order ≦ of SMSO. For example, the arrow with label “subClassOf” between the concept with label “patient” and another concept with label “human” shows that [human]≦[patient].

Furthermore, the set of arrows with label “domain” in Figure 2 defines *src* of SMSO, while another set of arrows with label “range” in Figure 2 defines *tagt* of SMSO. For example, the arrow with label “domain” between properties described by the rotundate rectangle with label “birth date” and another rotundate rectangle with label “human” shows that [human] ∈ *src*([birth date]). Therefore, since [human]≦[patient], it also holds that [patient] ∈ *src*([birth date]) by the property 5 in the definition of ontologies. Moreover, since Figure 2 has no concept *C* except [human] and [patient] that satisfies [human]≦*C*, it also holds that *src*([birth date]) = {[human], [patient]}. Similarly, one can obtain *tagt*([birth date]) = {[date]}.

Rectangles in the last two lines in Figure 2 describe values of SMSO. For example, rectangles with labels “stomach cancer”, “bowel cancer” and “lung cancer” describe values of [disease] by typ, respectively, which is not explicit in Figure 2.

**Definition (Ontology-Labeled Graph)** A quintuple (*N, E, src, tagt, L*) is a *graph labeled by an ontology* (*Conc, Val, Prop, Attr, src, tagt,* ≦) iff the following properties hold:

In the followings, both OR and NOT are fresh labels, that is, none of them belongs to *NL*∪*Prop*.

1. (*N, E, src, tagt*) is a graph.
2. *L* is a function of *N*∪*E* into *NL*∪*Prop*∪{OR*,* NOT} such that
  *L*(*e*) ∈ *Prop*∪{OR*,* NOT} for each *e*∈*E*.
3. For each *e* ∈ *E*, if *L*(*e*) ∈ *Prop* , then *L*(*src*(*e*)) ∈ *src*(*L*(*e*)) and *L*(*tagt*(*e*)) ∈ *tagt*(*L*(*e*)).
4. For each *e* ∈ *E*, if *L*(*e*) = NOT, there is no *e*_0_ other than *e* such that *src*(*e*_0_) = *src*(*e*) or *tagt*(*e*_0_) = *src*(*e*).
5. For each *e* ∈ *E*, if *L*(*e*) = OR, then *L*(*tagt*(*e*))≦*L*(*src*(*e*)).

A graph labeled by an ontology *O* is called an *O*-labeled graph, or simply a labeled graph.

Nodes, edges, paths, connectivity and subgraphs are defined over an ontology labeled graph *G* = (*N, E, src, tagt, L*) as *G* is regarded as a graph (*N, E, src, tagt*).

**Figure 3:**
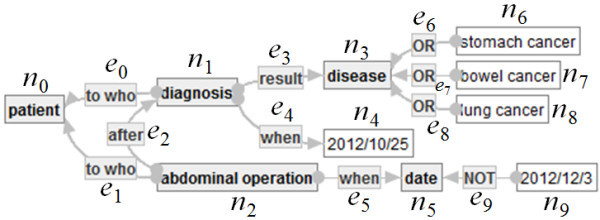
**SMSO-labeled graph*G***_**1**_**.**

**Example of an Ontology-Labeled Graph.** As an example of a SMSO-labeled graph, *G*_1_ = (*N*_1_*, E*_1_*, src*_1_*, tagt*_1_*, L*_1_) is defined in Figure 3.

The components of *G*_1_ are defined as follows.

1. *N*_1_ := {*n*_0_, *n*_1_, *n*_2_, *n*_3_, *n*_4_, *n*_5_, *n*_6_, *n*_7_, *n*_8_, *n*_9_}.
2. *E*_1_ := {*e*_0_, *e*_1_, *e*_2_, *e*_3_, *e*_4_, *e*_5_, *e*_6_, *e*_7_, *e*_8_, *e*_9_}.
3. *e*_0_:*n*_1_ → *n*_0_, *e*_1_:*n*_2_ → *n*_0_, *e*_2_:*n*_2_ → *n*_1_, *e*_3_:*n*_1_ → *n*_3_, *e*_4_:*n*_1_ → *n*_4_.
4. *L*_1_ is defined, as follows]
  *L*_1_(*n*_0_):=[patient], *L*_1_(*n*_1_):=[diagnosis], *L*_1_(*n*_2_):=[abdominal operation], *L*_1_(*n*_3_):=[disease], *L*_1_(*n*_4_):=[2012/10/25], *L*_1_(*n*_5_):=[date], *L*_1_(*n*_6_):=[stomach cancer], *L*_1_(*n*_7_):=[bowel cancer], *L*_1_(*n*_8_):=[lung cancer], *L*_1_(*n*_9_):=[2012/12/3],
  *L*_1_(*e*_0_):=[to who], *L*_1_(*e*_1_):=[to who], *L*_1_(*e*_2_):=[after], *L*_1_(*e*_3_):=[result] and *L*_1_(*e*_4_):=[when], *L*_1_(*e*_5_):=[when], *L*_1_(*e*_6_):= *L*_1_(*e*_7_):= *L*_1_(*e*_8_):= [OR], *L*_1_(*e*_9_):=[NOT].

**Definition (Negativeness and Positiveness)** An ontology *O* = (*Conc, Val, Prop, Attr, src, tagt,* ≦) and an *O*-labeled graph *G* = (*N, E, src, tagt, L*) are given.

1. An edge *e*∈*E* is *negative* if *L*(*e*) = NOT. Otherwise, the edge *e* ∈ *E* is *positive*.
2. A node *n*∈*N* is *negative* if *n* = *src*(*e*) for some negative *e* ∈ *E*. Otherwise, the node *n*∈*N* is *positive*.
3. The labeled graph *G* is *negative* if *L*(*e*) = NOT for some negative *e* ∈ *E*. Otherwise, the labeled graph *G* is *positive*.

The notation *PE*^*G*^ denotes the set of all the positive edges. The notation *PN*^*G*^ denotes the set of all the positive nodes. *PE*^*G*^ and *PN*^*G*^ are abbreviated to *PE* and *PN*, respectively.

**Definition (Strictness)** An ontology *O* = (*Conc, Val, Prop, Attr, src, tagt,* ≦) and an *O*-labeled graph *G* = (*N, E, src, tagt, L*) are given.

1. An edge *e*∈*E* is *strict* if *L*(*e*) ≠ OR.
2. A node *n*∈*N* is *strict* if each edge *e*∈*E* such that *tagt*(*e*) = *n* is strict.
3. The graph *G* is *strict* if all the edges are strict.

Definition (OR*-property) An ontology *O* = (*Conc, Val, Prop, Attr, src, tagt,* ≦) and an *O*-labeled graph *G* = (*N, E, src, tagt, L*) are given. For two nodes *n, n’* ∈ *N*, the property *n* → _OR*_n’ holds if the following properties hold:

1. *n* is strict.
2. There is a path (*e*_1_*, e*_2_*, …, e*_*m*_) with length *m*≧0 such that
  – *src*(*e*_1_) = *n*, *tagt*(*e*_*m*_) = *n’*,
  – *tagt*(*e*_*i*_) = *src*(*e*_*i*+1_) for *i* = 1, 2, *…,m-*1 and
  – *L*(*e*_*i*_) = OR for each *e*_*i*_.

Note that *n* → _OR*_n for each strict node *n* ∈ *N*.

**Definition (Disjunctive Embedding)** An ontology (*Conc, Val, Prop, Attr, src, tagt,* ≦) is fixed.

A connected strict labeled graph *G* = (*N, E, src, tagt, L*) and a connected labeled graph *G*’ = (*N’, E*’*, src*’*, tagt*’*, L*’) are given. A function *f*: *N*∪*E* → *N’*∪*E*’ is a *disjunctive embedding* of *G* into *G*’ if the following properties hold.

1. *f* is injective.
2. For each *n* ∈ *N*, *f*(*n*) is a strict node and *L*(*n*) = *L*’(*f*(*n*)).
3. For each *e* ∈ *E*, *f*(*e*) ∈ *E*’ and *L*(*e*) = *L*’(*f*(*e*)). Thus *f*(*e*) is strict.
4. For *e* ∈ *E*, *f*(*src*(*e*)) → _OR*_src(*f*(*e*)) and *f*(*tagt*(*e*)) → _OR*_tagt(*f*(*e*)).
5. For *n* ∈ *N* and *e*’ ∈ *E*’, if *f*(*n*) → _OR*_src(*e*’), then there is an *e* ∈ *E* such that *n* = *src*(*e*) and *e*’ = *f*(*e*).
6. For *n* ∈ *N* and *e*’ ∈ *E*’, if *f*(*n*) → _OR*_tagt(*e*’), then there is an *e* ∈ *E* such that *n* = *tagt*(*e*) and *e*’ = *f*(*e*).

If there is a disjunctive embedding of *G* into *G*’, then *G* is a *disjunct* of *G*’.

**Figure 4:**
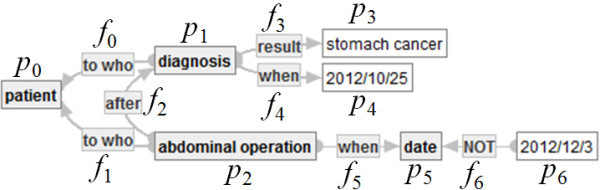
**Strict SMSO-labeled graph*G***_**2**_**.**

**Example of Disjunctive Embedding.** Let *G*_1_ be a connected SMSO-labeled graph indicated by Figure 3 and *G*_2_ = (*N*_2_*, E*_2_*, src*_2_*, tagt*_2_*, L*_2_) a connected strict SMSO-labeled graph in Figure 4 below.

Here, an injective function φ: *N*_2_∪*E*_2_ → *N*_1_∪*E*_1_ is defined, as follows.

– φ(*p*_*i*_): = *n*_*i*_ if *i* ∈ {0, 1, 2, 4, 5},
– φ(*p*_3_): = *n*_6_,
– φ(*p*_6_): = *n*_9_,
– φ(*f*_*i*_): = *e*_*i*_ if *i* < 6, and
– φ(*f*_6_): = *e*_9_.

Then, one can easily check that φ is a disjunctive embedding from *G*_2_ to *G*_1_.

**Definition (Terminologies on Labeled Graphs)** A labeled graph *G* = (*N, E, L, src, tagt*) is given.

1. For a concept name *C*, the set {*n* ∈ *N | C*≦*L*(*n*)} is called the *extension* of *C*.
2. For a property name *R*, the set {(*n*, *n* ’) ∈ *N* × *N*|*L*(*e*) = *Rand e* : *n* → *n* ’} is called the *extension* of *R*.
3. For an node *n* ∈ *N*, an attribute name *A* and a value *v* ∈ *Val*, if *e*: *n → n’* and *L*(*n’*) = *v* for some *e* ∈ *E* and *n’* ∈ *N*, then it is said that *v* is the *value* of *A* of *n*.

**Definition (Refinement)** An ontology *O* = (*Conc, Val, Prop, Attr, src, tagt,* ≦) and two strict labeled graphs *G* = (*N, E, L, src, tagt*) and *G*’ = (*N’, E*’*, L*’*, src*’*, tagt*’) are given. A function *f*: *PN*^*G*^∪*PE*^*G*^ → *N’*∪*E*’ is a *refinement* of *G* into *G*’ iff the following properties hold:

1. *L*(*n*)≦*L*’(*f*(*n*)) for each *n* ∈ *PN*_*G*_.
2. *L*’(*f*(*e*)) = *L*(*e*) for each *e* ∈ *PE*_*G*_.
3. For each *e* ∈ *PE*_*G*_ and *n, n’* ∈ *PN*_*G*_, if *e*: *n* → *n’*, then *f*(*e*): *f*(*n*) → *f*(*n’*).
4. For each negative *e* ∈ *E*, there is no *C* ∈ *Conc*∪*Val* such that *L*(*src*(*e*))≦*C* and *L’*(*f*(*tagt*(*e*)))≦*C*.

*f*: *G* → *G*’ denotes that *f* is a refinement of *G* into *G*’.

**Figure 5:**
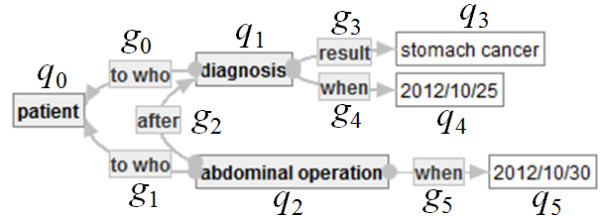
**Strict (positive) SMSO-labeled graph*G***_**3**_**.**

**Example of Refinement.** Let *G*_2_ = (*N*_2_*, E*_2_*, src*_2_*, tagt*_2_*, L*_2_) be a connected strict SMSO-labeled graph in Figure 4 and *G*_3_ = (*N*_3_*, E*_3_*, src*_3_*, tagt*_3_*, L*_3_) a connected strict positive SMSO-labeled graph in Figure 5 below.

Here, a refinement *ψ*: *PN*_*G*2_∪*PE*_*G*2_ → *N*_3_∪*E*_3_ is defined, as follows.

*ψ*(*p*_*i*_) := *q*_*i*_ and *ψ*(*f*_*i*_): = *g*_*i*_ if *i*≦5,

*ψ*(*p*_6_): = *q*_5_ and *ψ*(*f*_6_): = *g*_5_.

Then, one can easily check the following claim that *ψ* is a refinement from *G*_2_ to *G*_3_.

**Definition (Data Base)** An ontology *O* = (*Conc, Prop, Attr, Val, src, tagt,* ≦) is fixed. Then, a labeled graph *D* = (*N, E, L, src, tagt*) is a *data base* of *O* iff the following property holds.

– For each *n* ∈ *N with L(n)* ∈ *Conc* and for each *A* ∈ *attr* (*L(n)*), there is a unique *v* ∈ *Val* such that

For a data base *D* = (*N, E, L, src, tagt*), an *n* ∈ *N* with *L*(*n*) ∈ *Conc*, and for an *A* ∈ *attr* (*L*(*n*)), the notation *A*^*D*^(*n*) denotes the unique *v* ∈ *Val* such that ∃*e* ∈ *E, n’* ∈ *N. L*(*e*) = *A* & *L*(*n’*) = *v*. *A*^*D*^(*n*) is often abbreviated to *A*(*n*).

**Definition (Instance)** An ontology *O* and a data base *D* of *O* are fixed. Let *G* be a labeled graph, and *f* a refinement of *G* into *D*. Then, the image of *f* is called an *instance* of *G* in *D*.

**Figure 6:**
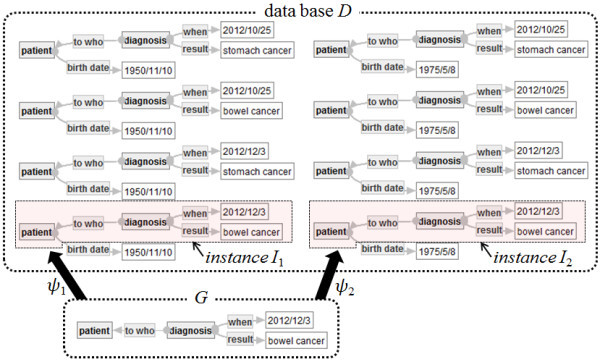
**Two refinements*ψ***_**1**_**and*ψ***_**2**_**of G into D and their instances*I***_**1**_**and*I***_**2**_**.**

**Example of data base and instances** Let *D* and *G* be a data base and a strict labeled graph in Figure 6, respectively. Note that *D* consists of eight connected graphs. There exist two refinements *ψ*_1_ and *ψ*_2_ of *G* into *D* and their instances *I*_1_ and *I*_2_, which are indicated by the two polygons with the dotted lines in Figure 6. Note that there is no instance of *G* in *D* other than *I*_1_ or *I*_2_.

### Object graph

**Notations on Labeled Graphs** For a labeled graph *G* = (*N, E, L, src, tagt*), *N*_*G*_, *E*_*G*_, *L*_*G*_, *src*_*G*_ and *tagt*_*G*_ denote *N*, *E*, *L*, *src* and *tagt*, respectively.

**Definition (Object Graph)** A pair (*r, G*) is an *object graph* of an ontology *O* iff *G* is a connected *O*-labeled graph and *r* ∈ *N*_*G*_. The component *r* of (*r, G*) is called the *root node* or the root.

**Definition (Subgraph of Object Graph)** For an object graph (*r, G*) and a subgraph G* with *r* ∈ *N*_G*_, (r, G*) is called a *subgraph* of (*r, G*).

**Definition (Strict Object Graph)** An object graph (*r, G*) is *strict* if *G* is strict.

**Definition (Disjunctive Embedding)** An object graph *X* = (*r, G*) and a strict object *Y* = (*r*’*, G*’) are given.

A function *f*: *N*_*G’*_∪*E*_*G’*_ → *N*_*G*_∪*E*_*G*_ is a *disjunctive embedding f*: *Y* → *X* if *f* is a disjunctive embedding *f*: *G*’ → *G* and *f*(*r*’) = *r*. If there is a disjunctive embedding *f*: *Y* → *X*, then *Y* is called a *disjunct* of *X*.

**Definition (Instantiation)** An object graph *X* = (*r, G*) and a database *D* are given. A pair (φ, *ψ*) is called an *instantiation* of *X* into *D* if there is a strict object graph *Y* = (*r*’*, G*’) such that the following properties hold:

– φ is a disjunctive embedding of *Y* into *X*.

– *ψ* is a refinement of *Y* into *D*.

Let *D* be a database of *O* and φ a morphism of *G* into *D*. Then φ(*r*) ∈ *ND* is called the *instance* of (*r, G*) in *D* by φ. The set {(φ^-1^(*r*)) *|* (φ, *ψ*) is an instantiation of (*r, G*) into *D*} is called the *extension* of (*r, G*) in *D*.

**Figure 7:**
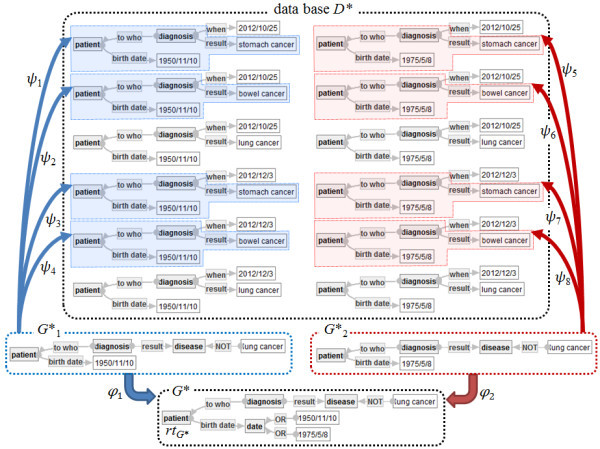
Extension of an object graph G* in a data base D*.

**Example of Extension of an object graph in a data base** Let D* and (*rt*, G*) be a data base and an object graph in Figure 7, respectively. Then, there exist two disjuncts G*_1_ and G*_2_ of G*. Moreover, G*_1_ has four refinements *ψ*_1_,…, *ψ*_4_ into D*, while G*_2_ has four refinements *ψ*_5_,…, *ψ*_8_ into D*. Thus, the extension of (*rt*, G*) in D* is {*ψ*_*i*_(*φ*_1_^− 1^(*rt*))|*i* = 1, …, 4} ∪ {*ψ*_*i*_(*φ*_2_^− 1^(*r*))|*i* = 5, …, 8}.

### Semantics of object graphs

**Definition (Assignment of an Object Graph)** An ontology *O* = (*Conc, Prop, Attr, Val, src, tagt,* ≦) is fixed. Then, an *assignment* α_*O*_ of *O* is a function from *Conc* ∪ *Prop* ∪ *Val* to a family of sets that satisfies the following properties:

1. α_*O*_ assigns to each *C* ∈ *Conc* a set *S*_*C*_.
2. α_*O*_ assigns to each *v* ∈ *Val* the singleton set {*v*}.
3. α_*O*_ assigns to each *P* ∈ *Prop* a relation *R*_*P*_ ⊂ (∪_*x*∈ *src*(*P*)_*α*_*O*_(*x*)) × (∪_*y*∈ *tagt*(*P*)_*α*_*O*_(*y*)).
4. For each *X*, *Y* ∈ *Conc* ∪ *Val*, if *X*≦*Y* then α_*O*_(*X*) ⊃ α_*O*_(*Y*).

**Definition (Interpretation of Object Graphs)** An ontology *O* = (*Conc, Prop, Attr, Val, src, tagt,* ≦), an assignment α_*O*_, and a data base *D* of *O* are fixed. Then, for an object graph (*r*, *G*) with *G* = (*N*, *E*, *L*, *src*, *tagt*), an interpretation [[(*r*, *G*)]] over (*O*, α, *D*) is defined, as follows.

1. If *G* is strict and *G* has no negative edge, then

where *N* = {*X*_0_, …, *X*_*n*_} and *r* = *X*_0_.

2. Otherwise, [[(*r*, *G*)]] := ∪ {[[(*ψ*(*φ*^− 1^(*r*)), *ψ*(*φ*^− 1^(*G*)))]]|(*φ*, *ψ*) is an instantiation of (*r, G*) into *D* }.

**Notation of Objective Graphs** An object graph (*r, G*) is often abbreviated to *G*, and (*r, G*) is often denoted by (*N, E, L, src, tagt*, *r*) instead of (*r*, (*N, E, L, src, tagt*)). the interpretation of (*r, G*) is denoted by just [[*G*]].

## Medical service ontology

This section defines *Medical Service Ontology (MSO)*. MSO provides vocabulary words for defining quality indicators. MSO is developed by an ontology developing tool called “Semantic Editor” (Hasida 2013).

In the following subsections, main concepts and their attributes are defined, and then main properties other than attributes are defined.

### Outline of MSO concept names and their attributes

To describe results of assessment of medical service quality, the following vocabulary words are especially important:

1. Patients and their states,
2. Medical services in hospitals to such patients, and
3. Outcomes of such medical services.

In many cases, an outcome is represented as an event that happens in a hospital (see Section 2.2 below). For example, death of a patient as an outcome of a surgery is represented by an event of a death discharge of a hospital. Therefore, concepts related to patients, states of patients and events in hospitals are regarded as main concepts in MSO. In the rest of this subsection, the main concepts are explained.

### Patients

Basic concepts related to patients and their attributes are defined, as follows.

**Figure 8:**
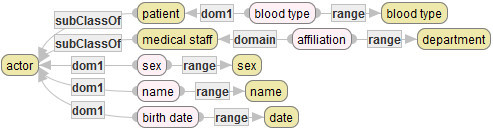
**Basic concepts and attributes related to patients.**

In Figure 8, yellow rounded rectangles denote concepts, and pink rounded rectangles denote attributes. In general, pink rounded rectangles in diagrams on Semantic Editor denote properties. This paper classifies properties between concepts into attributes of concepts and relations between concepts. The concept [patient] has attributes 〈blood type〉, 〈sex〉, 〈name〉, 〈birth date〉. The values of these attributes are supposed to be basically eternal for one patient.

In defining quality indicators, it is often useful to classify patients from the viewpoint of assessment of medical service quality. The following diagram classifies patients by the aspects of states of pregnancy and childbirth, a grown process, a degree of psychosomatic disorder, and positions as hospital staffs.

**Figure 9:**
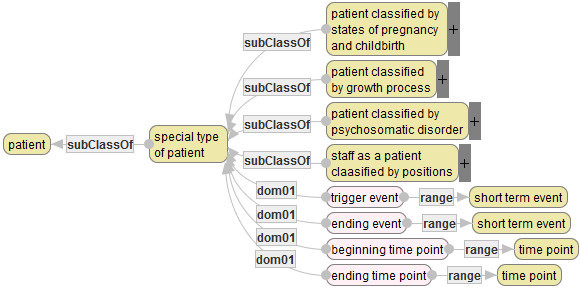
**Patients classified by four aspects (partial).**

A “+” mark in the diagram of Figure 9 denotes that there are sub-diagrams from the concepts (yellow rounded rectangles) with “+” marks and that the sub-diagrams are abbreviated due to limitation of space.

### Events

**Figure 10:**
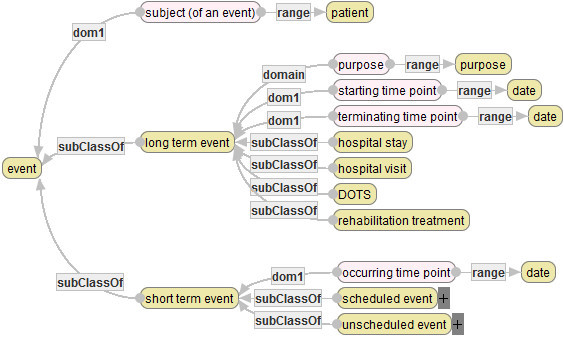
**Basis concepts and attributes related to events (partial).**

An event is defined to be what a medical staff or a hospital executes for a patient or what happens to him/her in a hospital. Events are classified into long-term events and short-term events, and short-term events are classified into scheduled events and unscheduled events (Figure 10).

A long-term event usually takes multiple days. For example, a hospital stay (a hospitalization) is a long-term event. Basically, a long-term event is executed by a medical staff or a hospital for a patient. On the hand, a short-term event does not basically take more than two days. For example, “admission”, “discharge”, “diagnosis”, “examination” are “operation (surgery)” are typical scheduled short-term events, while “death”, “falling” and “bone fracture” are typical unscheduled short-term events. In MSO, usual medical services are regarded as scheduled events, while accidents such as deaths are regarded as unscheduled events. Each typical event is furthermore classified into detailed classes. For example, examination events are classified into about thirty types of examinations.

Each long-term event has attributes the subject (target patient), purposes, the starting date and the ending date, while each short-term event has the subject, occurring time point (cf. Figure 10). Though scheduled and unscheduled events have their own attributes, the explanations are omitted due to limitation of space.

### States of patients

**Figure 11:**
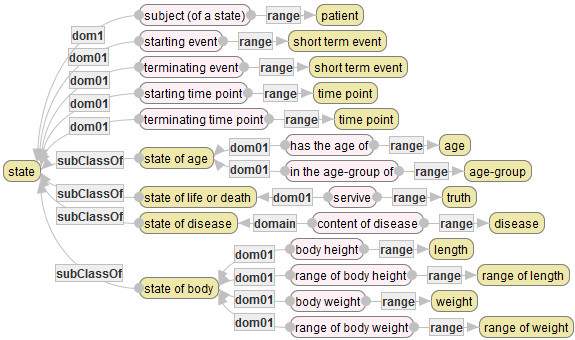
**Basis concepts and attributes related to states of patients.**

A state of patient denotes a health state or a condition of a patient at a time point. The diagram in Figure 11 defines main states: age, state of life or death, state of disease that a patient possesses, and basic body properties. These states are used to describe a feature of a patient as a target of a medical service or an outcome of a patient that cannot be represented by any event.

### Main relations in MSO

A property in MSO that is not an attribute is called a *relation*. The primary relations between concepts are defined, as follows.

*Relations of patients and events*: MSO has relations between the concept [patient] and concepts of events. For example, the following relation denotes the relations between patients and their hospital stays (a labeled angle bracket denotes a relation).

These relations describe relationships between events and the patients as subjects of the events. The explanation of the relations between patients and other events are omitted due to limitation of space.

*Relations of patients and states*: The relations are also defined between [patient] and concepts of patients’ states. For example, the following relation denotes the relationship between patients and their states of diseases.

These relations are defined to describe relationships between patients, types of diseases, degrees of the diseases, and the durations. Therefore, all concepts of patients’ states have the attributes of starting time points and terminating time points to make clear the durations of the states. The explanation of the relations between patients and other states are omitted due to limitation of space.

*Relations of time ordering*: The relations are also defined between the concepts of events and patients’ states. For example, the following relations denote the relationships between operations.

Here, “<p>” denotes a parameter. For example, the relation 〈before more than <2 weeks>〉 consists of a pair < op_*1*_, op_*2*_ > if op_*1*_ and op_*2*_ are performed and if op_*1*_ is performed more than two weeks before op_*2*_.

*Belonging relations of events*: The relations are defined between concepts of events with no term and events with terms. For example, the following relation denotes the relations between operations and hospital stays that have operations.

The relation contains a pair (op, sty) of an event of an operation op and that of a hospital stay sty if op is performed in the duration of sty.

In the following sections, only MSO is considered as an ontology and fix an assignment α of MSO and a data base *D* of MSO. Thus, in what follows, α and D are omitted from interpretations of MSO-labeled object graphs.

## Quantifying concepts

A *quantifying concept* describes a way to quantify the target as a function that has an object graph and optional parameters as input data and that outputs a numerical value. In general, one can classify quantifying concepts into three types. In the following, each type of quantifying concept is introduced. A quantifying concept is denoted by 〈name of a quantifying concept〉. Note that a concept is usually identified with its extension and that the extension is assumed to be finite.

### Total numbers

For a finite set *S*, the summation of numbers obtained from elements of *S* is called the total number of *S*. For example, if each element is assigned to 1 as the existence of the element, then the total number is the same as the cardinality of *S*, that is, the number of elements of *S*. The quantifying concept ⋘cardinality⋙ is regarded as a function that has an object graph  as input data and that outputs the cardinality of .

For a concept *S* and attributes *A*_*1*_,…, *A*_*n*_ of *S*, a real-valued function on the product set of (extensions of) *A*_*1*_,…, *A*_*n*_ is called an *attribute quantifying function*. Moreover, for a concept *S*, attributes *A*_*1*_,…, *A*_*n*_ of *S*, and an attribute quantifying function *f* of *A*_*1*_,…, *A*_*n*_, the summation Σ _s∈S_*f*(*s.A*_*1*_,…, *s.A*_*n*_) is called the *total attribute number* of *S* with respect to *A*_*1*_,…, *A*_*n*_ and *f*, where *s.A*_*i*_ denotes the value of an instance *s* with respect to *A*_*i*_.

The quantifying concept ⋘ total attribute number ⋙ is regarded as a function that has the following data as input data:

1. an object graph ,
2. attributes *A*_*1*_*,…, A*_*n*_ of (the label of) the root node  of , and
3. an attribute quantifying function *f* of *A*_*1*_*,…, A*_*n*_.
  Moreover, ⋘total attribute number ⋙ outputs the total attribute number of  with respect to *A*_*1*_,…, *A*_*n*_ and *f*.

### Rate

For a finite set S and a subset S* of S, the rate of the total number of S* among the total numbers of S obtained in the same way as that to calculate the total number of S* is called a rate of S* among S. In particular, the rate of the cardinality of S* among that of S is called the cardinality rate of S* among S. Moreover, the rate of the total attribute number of S* with respect to *A*_*1*_*,…, A*_*n*_ and *f* among that of S with respect to the same attributes and the same attribute quantifying function is called the total attribute number rate.

The quantifying concept ⋘cardinality rate⋙ is regarded as a function that has the following data as input data:

1. An object graph , and
2. A subgraph  of .

In contrast, the quantifying concept ⋘ total attribute number rate ⋙ is regarded as a function that has the following data as input data:

1. an object graph ,
2. a subgraph  of ,
3. attributes *A*_*1*_*,…, A*_*n*_ of , and
4. an attribute quantifying function *f* of *A*_*1*_*,…, A*_*n*_.

Moreover, ⋘total attribute number rate⋙ outputs the rate of the total attribute number of  with respect to *A*_*1*_*,…, A*_*n*_ and f among that of  with respect to the same attributes and the same attribute quantifying function.

### Average

For concept S, attributes *A*_*1*_*,…, A*_*n*_ of S, and attribute quantifying function f, the ratio of the total attribute number of S with respect to *A*_*1*_*,…, A*_*n*_ and f and the cardinality of S is called the average of the value of S with respect to *A*_*1*_*,…, A*_*n*_ of *f*. The quantifying concept ⋘average⋙ is regarded as a function that has the same input data as that of ⋘total attribute number⋙ and that outputs the average of the value of S with respect to *A*_*1*_*,…, A*_*n*_ of *f*.

### Ontology of quantifying concepts

Quantifying concepts that are explained in the previous subsections are organized, as follows.

The graphs above show classifications and main attributes of quantifying concepts from the three viewpoints. The first graph classifies quantifying concepts according to the definitions in Sections 6.1, 6.2 and 6.3. The second graph classifies quantifying concepts into those having a single object graph and those having two object graphs. Finally the third graph classifies quality indicators into those having only (an) object graph(s) and those having (an) object graph(s) plus attribute of the root concept and an attribute quantifying function. The latter quantifying concepts are called *attribute-based quantifying concepts (abqc)*.

**Figure 12:**
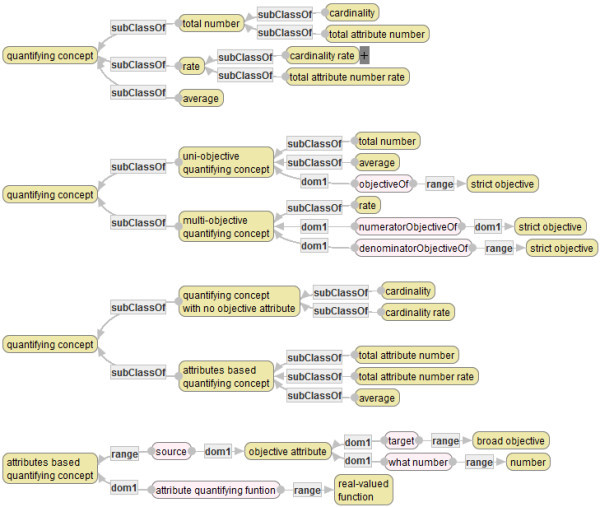
**Ontology of Quantifying Concepts (partial).**

The fourth graph shows special attributes of abqcs. There are two types of special attributes of abqcs. The first type of a special attribute indicates an attribute of a target of the quantification, and the second type of a special attribute indicates an attribute quantifying function. Note that the range of an objective attribute is not a target attribute itself but the range of it, because each instance of a quantifying concept (in the ontology in Figure 12) is used to be a part of a quality indicator graph (cf. the next section).

## Quality indicators in QI-RS

### Quality indicator graph

In this section, a graph-based representation of a quality indicator is defined. It is called a *quality indicator graph*. A quality indicator graph consists of one or two MSO-labeled object graphs and a quantifying concept.

Definition of quality indicator graphs needs to extend attributes of a concept name in an ontology. Roughly speaking, an extended attribute of a concept name *C* is an attribute of some concept name that has a relation with C. For example, if an event “operation” has a relation “is-an-event-within” with another event “hospital stay”, one can consider an extended attribute “operation-executed-date” of the “hospital stay” that is actually an attribute of “operation”. By using such an extended attribute, one can define a special quality indicator such as “an average of the lengths of hospital stays after their operations”.

**Definition (Extended Attribute)** For an object graph , if there exists a strict subgraph *S* of G such that *PN*S contains the root node *rt* of , then, an attribute of a concept node in *PN*^*S*^ is called an *extended attribute* of *L*(*rt*) over .

**Definition (Quality Indicator Graph)** Let *Q* be a quantifying concept,  an object graph, *A*_*1*_,…, *A*_*n*_ (*n*≧0) extended attributes of  in , and φ an attribute quantifying function of *A*_*1*_,…, *A*_*n*_. Then, a labeled graph  is defined based on *Q*, , *A*_*1*_,…, *A*_*n*_ and φ, according to the following cases.

**Case A.** If *Q* is a total number type of quantifying concept, then  is defined, as follows.

(i). ,^a^ where

(i-i) *m* = 0 if *n* = 0, and

(i-i) *m* = 1 otherwise.

(ii). 

(iii). , where

(iii-i) 

(iii-i) *f*_*i*_: *_0_ → *_i_. (*i* = 1,…, *n*) and

(iii-i) *f*_*n+m*_: *_0_ → *_n+m_ if *m* = 1.

(iii-i) .

(iv). The label function  is defined, as follows.

(iv-i) .

(iv-i)  = the range concept of *A*_*i*_ (*i* = 1,…,*n*)

(iv-i) 

(iv-i)  (See the second graph in Figure 12.)

(iv-i)  (See the third graph in Figure 12.)

(iv-i) . (See the third graph in Figure 12.)

(iv-i) 

(iv-i)  if *x* is a node or an edge of .

**Case B.** If *Q* is a rate type of quantifying concept, then, for a subgraph * of , ℚ is defined, as follows:

(i)., where
  (i-i) *m* = 0 if *n* = 0, and
  (i-ii) *m* = 1 otherwise.
(ii)..
(iii)., where
  (iii-i),
  (iii-ii),
  (iii-iii) *f*_*i*_: *_0_ → *_i_. (*i* = 2,…,*n* + 1) and
  (iii-iv) *f*_*n+m+1*_: *_0_ → *_n+m+1_ if *m* = 1.
  (iii-v). (*i* = 1,…,*n*).
(iv). The label function *L*_ℚ_ is defined, as follows:
  (iv-i).
  (iv-ii) = the range concept of *A*_*i*_ (*i* = 1,…,*n*)
  (iv-iii).
  (iv-iv) = the attribute “numeratorObjectiveOf” of *Q*. (See the second graph in Figure 12.)
  (iv-v) = the attribute “denominatorObjectiveOf” of *Q*. (See the second graph in Figure 12.)
  (iv-vi) = the attribute “*i*-th objective attribute” of *Q* (*i* = 2,…, *n* + 1). (See the fourth graph in Figure 12.)
  (iv-vii) = the attribute “attribute quantifying function” of *Q*. (See the fourth graph in Figure 12.)
  (iv-viii).
  (iv-ix) if *x* is a node or an edge of .

**Case C.** If *Q* is the average,  is defined in the same way as that of Case A.

 in Definition 7 is called a *quality indicator graph*, and  the *main object graph* of .

### Examples of quality indicator graphs

Two examples of quality indicator graphs are shown in this subsection.

**Figure 13:**
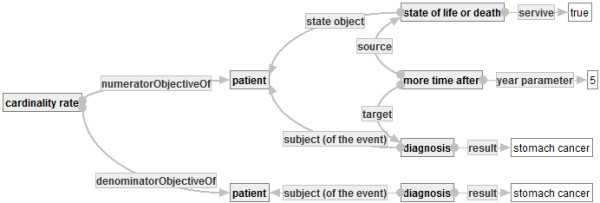
**Quality indicator graph of the rate of 5-year surviving stomach cancer patients.**

**Example of Quality Indicator Graph** One can develop a quality indicator “the rate of 5-year surviving stomach cancer patients” as a quality indicator graph in Figure 13. The graph consists of the quantifying concept ⋘ cardinality rate ⋙ with attributes “numeratorObjectiveOf” and “dinomiratorObjectiveOf” and two object-graphs.

**Example of Quality Indicator Graph (2)** Another quality indicator graph “Average post-operative length of hospital stay for stomach cancer” is developed based on the quantifying concept ⋘average⋙. The calculation formula CF2 is the average length of hospital stays after the operations of gastrectomy, partial gastrectomy or laparoscopic assisted distal gastrectomy (LADG), where the patients admit on the grounds of stomach cancers. CF2 is represented to be a quality indicator graph , as follows.

### Semantics of quality indicator graphs

**Definition (Instance and Extended Value)** Recall that an assignment α of MSO and a data base *D* of MSO have been fixed. Thus, for any concept *C* in MSO, the set α(C) is assigned. An element of α(C) is called an *instance* of *C*. For an object graph , a concept name *C* on , an extended attribute *A* of *C* over , and for an instance *c* of *C*, the *extended value c.A* of *c* with respect to *A* over  is the value of *c* by the composition α(*P*_1_)⋯α(*P*_n_) of functions α(*P*_1_),…, α(*P*_*n*_), where {*P*_1_,…, *P*_*n*_} is a path from *C* to *tagt*(*A*) over  that includes *A*.

**Figure 14:**
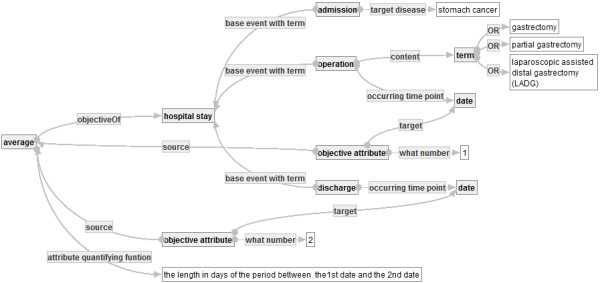
**Average post-operative length of hospital stays for stomach cancer.**

**Example of Extended Value** in Figure 14 has extended attributes of [hospital stay] based on [operation] and [discharge], respectively. Assume that the fixed assignment α assigns to each concept names and property names the set of natural things, for example, α assigns to the concept name [date] a set of dates. Then, extended values of a hospital stay *h* with respect to the extended attributes are the dates of the operation and the discharge in *h*, respectively.  also has an attribute quantifying function that calculates the length in days of the period between the extended values.

**Definition (Interpretation of Quality Indicator Graphs)** For a quality indicator graph , an interpretation  of  is defined, as follows.

**Case A1.** If the label of  is < cardinality>, then , where  is the object graph indicated by the edge from *rt*_ℚ_ with label “objectiveOf” and  is the cardinality of .

**Case A2.** If the label of  is a total attribute number, then , where

(i). and  is the main object graph,
(ii). *A*_*i*_ is the extended attribute of  that is indicated by the edge from  with label the “*i*-th objective attribute”,
(iii). *f* is the attribute quantifying function that is indicated by the edge from  with label “attribute quantifying function”, and
(iv). *s*.*A*_*i*_ is the extended value of an element *s* ∈ *S* with respect to *A*_*i*_ for *i = 1,…,n*.

**Case B1.** If the label of  is a cardinality rate, then , where  and  are the object graph and a subgraph of it that are indicated by the edges from  with labels “numeratorObjectiveOf” and “objectiveOf”, respectively.

**Case B2.** If the label of  is a total attribute number rate, then , where , and  and  are the object graphs indicated by the edges with labels “numeratorObjectiveOf” and “objectiveOf”, respectively. Moreover, *A*_*1*_,…,*A*_*n*_*, f* and *s.A*_*1*_,…, *s.A*_*n*_ are obtained from the same way as those of (ii), (iii) and (iv) of Case 2 above, respectively.

**Case C.** If the label of  is an average, then , where *S*, , *A*_*1*_,…,*A*_*n*_*, f* and *s.A*_*1*_,…, *s.A*_*n*_ are obtained from the same way as those of (i)-(iv) of Case A2.

## Evaluation of QI-RS

In this section, the authors show expressiveness of QI-RS by using existing quality indicators in (Donabedian 1980; Nihon Hospital Organization 2009; OECD 2006) and description patterns of medical services assessment criteria.

### Evaluation of QI-RS through re-defining existing quality indicators

To evaluate expressiveness of QI-RS, the authors re-developed all existing quality indicators in (Nihon Hospital Organization 2009) by using QI-RS. (Nihon Hospital Organization 2009) consists of 33 quality indicators. They verified that QI-RS could define all quality indicators in (Nihon Hospital Organization 2009) with no artifice. Moreover, they verified that QI-RS actually had the ability to define special vocabulary words. To show the points above, the authors re-define the quality indicator CF1 in what follows. Here, CF1 is an existing quality indicator in (Nihon Hospital Organization 2009), which is explained in Section 1.

The first purpose of this subsection is to define a graph that represents “hospital stays of patient aged 75 or over”. According to the definition of CF1 in Section 1, one can develop the graph in a rigorous manner, as follows.

**Figure 15:**
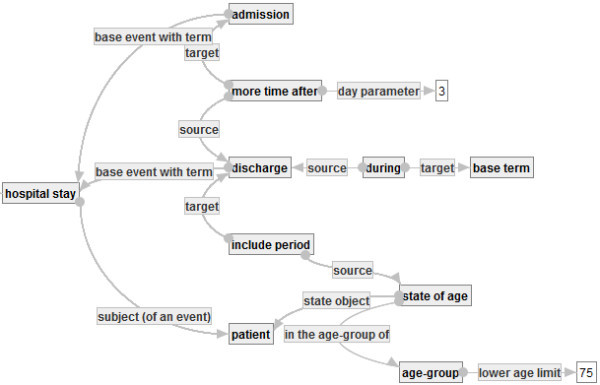
**: Hospital stays of aged patients.**

Let  be the graph in Figure 15. Then,  will be a segment that is contained in the quality indicator graph representing CF1. So, one may consider that the main objective graph  of CF1 is too large to develop directly. Therefore, to develop , it is useful to develop small graphs. Thus, in what follows, several small graphs are developed.

**Figure 16:**
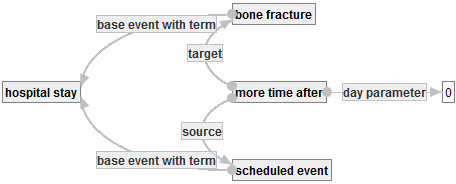
**: Hosptial stays in which inpatients break their bones and receive some scheduled events.**

The first small graph  is defined, as follows.

 in Figure 16 means the set of hospital stays in which inpatients break their bones and receive some schedule events after bone fractures.

The second small graph  that describes “treatment for fractures” is defined, as follows.

Note that the phrase “treatment for fractures” appears in CF1 but is not given any definition in CF1. It is not unusual that one can find ambiguities of several phrases only by trying to describe the phrases in a rigorous manner. QI-RS helps users find such ambiguities.

**Figure 17:**
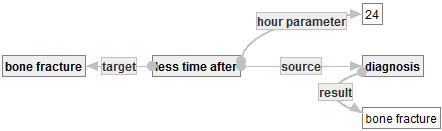
**: Special definition of bone fracture.**

Then, one of the authors re-defined the event of bone fracture in Figure 17. One may define the event of bone fracture as an accident of bone fracture plus a diagnosis with result bone fracture. (In fact, there exists a similar definition of bedsore.) In such a case, one can develop a graph  to define the event of bone fracture, as follows.

**Figure 18:**
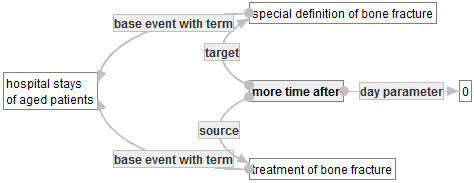
**: Main objective graph for representing CF1.**

By using , ,  and , one can develop the main objective graph  in Figure 18 that represents the set of hospital stays of patients aged 75 or over in which they break their bones and receive treatments for their bone fractures, as follows.

This graph is obtained from  by substituting , , and G* into , that is,

where BF, SE and HS are concept occurrences denoted by the nodes with labels ”bone fracture”, ”scheduled event” and ”hospital stay” in .

**Figure 19:**
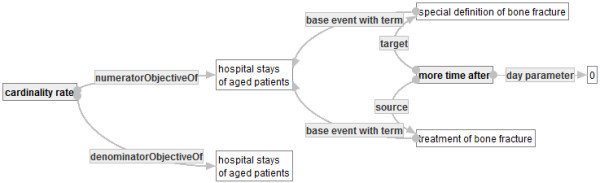
**: Quality indicator graph representing CF1.**

Finally one can develop a quality indicator graph ℚ in Figure 19 that represents CF1 by using  and , as follows.

Then, Q is ensured to be a quality indicator graph. By using substitution, one can develop a complicated quality indicator graph such as G incrementally. Moreover, the example shows the graph-based representation can address the problem explained in Section 1. And it shows that QI-RS realizes a representation of a quality indicator that satisfies both of formality and understandability.

**Figure 20:**
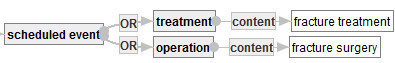
**: treatment of bone fracture.**

The word “treatment of fracture patients” in the calculating formula CF1 is not defined and has a vague meaning. One can define such a word as an object graph such as that in Figure 20. By developing such an object and sharing it among users, other uses can re-use it with explicitly checking its meaning. Moreover, a quality indicator “positive ratio of HIV to in-patients” in (Nihon Hospital Organization 2009) contains a phrase “HIV-positive in-patients”. The phrase is also ambiguous. In fact, to make clear the meaning of the phrase, one has to clarify from what the fact that a patient is HIV-positive is obtained. QI-RS prevents users from making such an ambiguous phrase, since QI-RS enforces uses to input data into necessary attributes of concepts that are used to define a new word or phrase.

In (Nihon Hospital Organization 2009), there are two quality indicators: “the acceptance rate of super severely retarded children” and “the acceptance rate of short-term hospitalizations of severely retarded children”. While the former is the ratio of the numbers of some patients, the latter is the ratio of the number of some patients to the number of some beds. Such an approximate calculation is sometimes necessary for convenience of calculation. However, it becomes difficult to fairly compare medical service qualities among hospitals if one often uses such an approximation unconsciously. The method to construct quality indicators based on quantifying concepts prevents one from abusing unnatural ways to construct quality indicators. Moreover, if one can unify the method to construct calculating formulas quality indicators for a given purpose, he/she can assign to suitable names them in a coherent manner.

### Evaluation of QI-RS based on expression patterns of assessment criteria of medical services

To evaluate expressiveness of QI-RS from the more general viewpoint, the authors constructed expression patterns of assessment criteria of medical services as follows:

1. What types of medical staffs and equipment does the hospital have?
2. What types of patients has the hospital treated?
3. What treatments has the hospital given to the patients?
4. What results has the hospital obtained, after the treatments?

The pattern is called Medical Service Assessment Description Pattern (MSADP).

And they classified existing quality indicators in (Fukui 2009; Nihon Hospital Organization 2009; OECD 2006) according to the description patterns above. Then, the following table was obtained:

Table 2 implies that there are 137 quality indicators that can be assigned to one of the four patterns, while 24 one that cannot be assigned to any pattern, and hence, about MSADP covers about 85% of quality indicators in [4] + [17] + [18].

**Table 2:** **Classification of quality indicators according to expression patterns**

	Pattern 1	Pattern 2	Pattern 3	Pattern 4	Not Pattern	Total
QIs in [4]	4	2	35	50	**20**	111
QIs in [17]	0	3	7	22	**1**	33
QIs in [18]	0	1	7	6	**3**	17
[4] + [17] + [18]	4	6	49	78	**24**	161

Next, the authors checked whether or not one could re-define each quality indicator classified according to MSADP by using QI-RS. Then, they obtained the result that one could re-define 134 quality indicators over 137 quality indicators by using QI-RS, and hence, QI-RS covered about 98% of quality indicators classified according to MSADP. Thus, one can believe that QI-RS have high expressiveness from the general viewpoint so that QI-RS covers more than 80% of existing quality indicators.

## Related works

Research of quality indicators has long history, and one can see a starting point in Nightingale’s work (Nightingale 1859). One can see researches on the ways to define quality indicators in (Collopy 2000; Donabedian 1980; Ito 2003; Mainz 2003; Montalto et al. 1999). Moreover, in recent years, comparison results of quality indicators among multiple hospitals or countries (Mainz et al. 2009; Mainz et al. 2004) and [ORCD]. Though these researches are important for actual definition of quality indicators for comparison of medical service quality, these researches have been done from the viewpoints of epidemiology. On the other hand, this paper focuses on how to describe quality indicators from the viewpoint of knowledge representation, especially, this paper focuses on a representation of quality indicator that satisfies understandability and formality.

Formality and understandability of ontology-based representation for medical services have been researched in (Huser et al. 2010; Mabotuwana & Warren 2009). The authors of (Mabotuwana & Warren 2009) propose a framework to indentify hypertensive patients who satisfy evidence-based criteria for quality improvement potential. They propose three issues for domain-modelling: (i) shareability, (ii) extensibility, and (iii) easy visualization of knowledge base for domain-modelling. On the other hand, the authors of (Huser et al. 2010) establish a query system of electoric health record data based on flowchart that indicates processes to treat patients. The authors propose an trade-off problem of readability and expressiveness of query representation. The authors of (Huser et al. 2010; Mabotuwana & Warren 2009) focus on how to represent queries correctly and/or easily on the basis of considerably restricting the domain of the querie, and their approaches are not easy to extend for evaluation of general medical service quality. This paper enhances formality and understandability of QI-RS by MSO that provides sufficient vocabulary words to define quality indicators and by establishing a general framework of ontology-based graph representation.

For more general medical information, there are a lot of researches for ontology-based information retrieval, or ontology-based information integration (for example, see (Hartel et al. 2005; Kaiser et al. 2007; Serban et al. 2007)). However, to define quality indicators, it is important to provide a sufficient vocabulary not only to represent concepts in medical domain but also to cover description patterns of medical service assessment such as “how a certain medical service was executed?” or “what results were obtained from a medical service?”. This paper provides MSO and object graphs, by which the authors specify description patterns of medical service quality assessment, and quantifying concepts, by which they stipulate how to quantitatively-represent medical service quality.

There also have been researches on graph-based knowledge representations (Chein & Mugnier 2009; Sowa 1984; Sowa 2008). The authors mainly have been focusing on the power of expression from the viewpoint of mathematical logic and computations of their representations. On the other hands, this paper focuses on compatibility of formality and understandability of QI-RS.

## Conclusion

This paper introduces a representation system QI-RS of quality indicators, towards development a framework to define quality indicators and calculate their values based on medical databases automatically. QI-RS represents a quality indicator as a labeled graph consisting of one or two object graph(s) based on Medical Service Ontology (MSO) and a quantifying concept. An object graph denotes a target of quantification, while a quantifying concept indicates a way to quantify the object graph. By constructing object graphs and quantifying concepts, the target and the way to quantify medical service quality independently. Moreover, MSO and quantifying concepts unify vocabulary words and constructions of quality indicators. By using MSO, object graphs and quantifying concepts, one can obtain graph-based representation of quality indicators that satisfies formality and understandability.

In the last half of this paper, the authors develop a library of all quality indicators in (Nihon Hospital Organization 2009) in QI-RS to show that QI-RS has sufficient expressiveness to develop actual quality indicators. To show expressiveness of QI-RS from the more general viewpoint, they also classified all quality indicators in (Fukui 2009; Nihon Hospital Organization 2009; OECD 2006) according to Medical Service Assessment Description Patterns (MSADP) and checked that most (98%) of quality indicators classified according to MSADP could be re-defined in QI-RS (Section 8.2).

## Endnote

^a^ The symbol “*i“ is used to denote an element of a set in this paper.

## Abbreviations

QI-FW: Framework to develop Quality Indicators and to calculate their values
QI-RS: Representation System of Quality Indicators
MSO: Medical service ontology
MSADP: Medical service assessment description patterns
GDM: Global data model
SE: Semantic editor.
